# Functionalized Nanocellulose Drives Neural Stem Cells toward Neuronal Differentiation

**DOI:** 10.3390/jfb12040064

**Published:** 2021-11-22

**Authors:** Sahitya Chetan Pandanaboina, Ambar B. RanguMagar, Krishna D. Sharma, Bijay P. Chhetri, Charlette M. Parnell, Jennifer Yanhua Xie, Malathi Srivatsan, Anindya Ghosh

**Affiliations:** 1 Department of Biological Sciences and Arkansas Biosciences Institute, Arkansas State University, Jonesboro, AR 72401, USA; drpandana@gmail.com (S.C.P.); krishna.sharma@smail.astate.edu (K.D.S.); 2 Department of Chemistry, University of Arkansas at Little Rock, Little Rock, AR 72204, USA; abrangumaga@ualr.edu (A.B.R.); bpchhetri@ualr.edu (B.P.C.); cmparnell15@yahoo.com (C.M.P.); 3 Department of Basic Sciences, New York Institute of Technology College of Osteopathic Medicine, Arkansas State University, Jonesboro, AR 72401, USA

**Keywords:** nanocellulose, rat fetal stem cells, neuronal differentiation, extracellular matrix

## Abstract

Transplantation of differentiated and fully functional neurons may be a better therapeutic option for the cure of neurodegenerative disorders and brain injuries than direct grafting of neural stem cells (NSCs) that are potentially tumorigenic. However, the differentiation of NSCs into a large population of neurons has been a challenge. Nanomaterials have been widely used as substrates to manipulate cell behavior due to their nano-size, excellent physicochemical properties, ease of synthesis, and versatility in surface functionalization. Nanomaterial-based scaffolds and synthetic polymers have been fabricated with topology resembling the micro-environment of the extracellular matrix. Nanocellulose materials are gaining attention because of their availability, biocompatibility, biodegradability and bioactivity, and affordable cost. We evaluated the role of nanocellulose with different linkage and surface features in promoting neuronal differentiation. Nanocellulose coupled with lysine molecules (CNC–Lys) provided positive charges that helped the cells to attach. Embryonic rat NSCs were differentiated on the CNC–Lys surface for up to three weeks. By the end of the three weeks of in vitro culture, 87% of the cells had attached to the CNC–Lys surface and more than half of the NSCs had differentiated into functional neurons, expressing endogenous glutamate, generating electrical activity and action potentials recorded by the multi-electrode array.

## 1. Introduction

In neurodegenerative diseases or trauma to the central nervous system (CNS), loss of neurons leads to irreversible damage that can significantly affect sensory and motor functions [1,2,3,4]. Neuronal death leads to a permanent loss of function, as fully differentiated neurons do not divide to produce new neurons. Researchers have been exploring several strategies for treating neurodegenerative diseases and injuries to the brain. An obvious solution to this would be to replace the damaged and dead neurons with healthy, new neurons to promote the recovery of lost functions. Neural stem cells (NSCs) have been used to provide cell-based therapies to address neurodegenerative diseases and trauma-induced function loss [5,6,7]. The biggest hurdle for this strategy so far is the tumorigenicity from the transplanted NSCs [8], because stem cells retain their ability to proliferate [9], and also because of the uncertainty regarding whether cells differentiate into glial cells or neurons [10]. Therefore, the transplantation of already differentiated neurons exhibiting neuronal electrical activity may be a better therapeutic option. However, this requires a large pool of differentiated neurons readily available in tissue banks (in vitro) for transplantation. Under in vitro conditions, the differentiation of sufficient neurons from NSCs has been challenging. The extracellular matrix (ECM) and secreted molecules are the main players that affect cell behavior, including differentiation of stem cells into mature cells [11,12,13,14]. Therefore, research is currently in progress to determine if fabricated culture surfaces could help promote the differentiation of NSCs mostly into neurons.

Nanomaterials have been widely used to manipulate cell behavior due to their nano-size, excellent physicochemical properties, ease of synthesis, and versatility in surface functionalization [15,16,17]. In designing and fabricating ECM networks, nanomaterial-based scaffolds and synthetic polymers have been widely used to simulate the micro-environment of the ECM in vivo [18,19,20]. Among them, cellulose is being used due to its wide availability, excellent biocompatibility, biodegradability, bioactivity, and regeneration-promoting features [21,22,23,24,25]. As a natural carbohydrate, cellulose is biocompatible and does not cause any allergic reactions in vivo [26]. It is broken down into shorter polysaccharides or sugar molecules and generally does not cause any toxicity unless inhaled into the lungs [26,27]. The cellulose found in bacteria and plants can be restructured into nanofibers, i.e., nanocellulose, which is a widely used natural polymeric material for various applications, including biomedical applications [27,28,29]. Moreover, networks of cellulose nanofibers have been synthesized for various biomaterial applications by crosslinking 2,2,6,6-tetramethyl−1-piperidinyloxy (TEMPO) oxidized cellulose nanofibers with other polymers, including poly(acrylic acid) [30,31]. Among the various forms of nanocellulose, biocompatible cellulose nanocrystal or crystalline nanocellulose (CNC) has been used as a novel and advanced nanomaterial in biomedical sciences [32,33,34,35].

It is well established that the properties of a culture surface can affect cells’ migration, adhesion, and morphology [36]. Cellulose of nanoscale size (1–100 nm) exhibits altered properties of the material, such as morphology, size, and composition, which could impact the adhesion, biocompatibility, growth, and development of cells [37,38]. Moreover, it is known that other properties of a substrate, such as stiffness, roughness [39], and surface charge [40,41,42], can influence the differentiation of stem cells. The robustness of CNC toward chemical or thermal treatment during sterilization processes is one of its advantages for serving as a cell culture substrate, because surfaces need to be sterilized before cell culture. The nanofibrillar structure of CNC, which can have a high water content, can maintain mechanical stability, and can mimic the microenvironment of the ECM, has been used for the differentiation of different cell types such as human hepatic cells [43] and fibroblasts [44].

The molecules that comprise the ECM in mammals have been reported to be of a nanometer scale (e.g., the 66 nm repeat banding of collagen fibers). Therefore, by mimicking the nanoscale topographic features of the ECM during biomaterial fabrication, CNC could plausibly be adapted to induce the same effects as the ECM [36,45]. Mammalian cells do not readily attach to cellulose due to its lack of integrin binding sites [43,44,46] and natural hydrophilic property with low non-specific protein adsorption [46,47]. Therefore, it is important to modify the surface properties of cellulose before it can be used as a substrate for NSC differentiation. The surface of CNC contains plenty of hydroxyl groups that allow for the incorporation of functional molecules to enhance cell adhesion and control the cell fate by modulating the interaction between CNC materials and cells/tissues [48]. Unlike normal cellulose, CNC has a fibrillar structure mimicking the ECM environment, which exhibits robustness toward chemical and thermal treatment and has hydroxyl groups aiding in surface modifications. Hence, it was selected as the basic material for surface modification in this study. We covalently linked CNC with lysine molecule (CNC–Lys) to confer positive charges that would enhance the attachment of the cells, and we tested them in vitro for rat NSC (rNSC) attachment, growth, and differentiation into neurons.

In addition to the differentiation phenotype, we further characterized the functionality of the differentiated neurons regarding the neurotransmitters and spontaneous firing activities they expressed. The generation of spontaneous electrical activity or firing of cells in vitro and in vivo is a characteristic feature exhibited by neurons during development. Multi-electrode arrays (MEA) have been an indispensable tool for non-invasive extracellular electrical recordings or stimulation of cells in vitro [49,50], both temporally and spatially [51]. On MEAs, cells can be cultured for several weeks or even months (long-term cultures) [52,53,54,55,56,57], and electrical activity can be assessed repeatedly throughout the culture period, as well as among multiple sites simultaneously, to characterize the interaction among cells. Local field potentials (LFPs) and extracellular action potentials (EAPs) from a population of neurons on a millisecond time scale are recorded using MEA. The recording of neuronal activity using dissociated neuronal cultures on MEAs has been reported by Pine et al. [58], and has since been employed in investigations of toxins, pharmacological compounds, and neuronal network connectivity using brain slices and stem cell cultures [59]. Herein, we used the same method to assess the functional activities of the neurons differentiated from rNSCs. In this research work, we used a renewable, biocompatible, and bioactive material, i.e., CNC, and chemically modified it with lysine for the first time to imitate the environment of the ECM. Our data indicate that CNC–Lys indeed promoted the differentiation of rNSCs into neurons during the three-week experimental period, and the differentiated neurons were electrically active, as indicated by the electrophysiological data recorded via MEA and reported here.

## 2. Results

### 2.1. Characterization of the CNC–Lys Material

**Figure 1:**
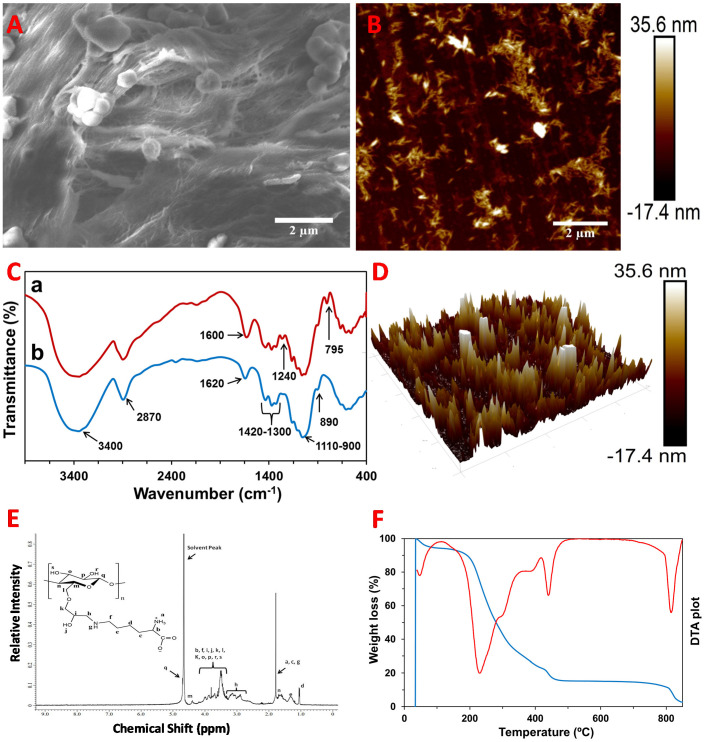
Surface morphology and structure analysis of the CNC–Lys substrate. SEM image with (**A**) ×10,000 magnification, showing the roughness and fibrous structure. AFM images, (**B**) height image, and (**D**) 3D image of the CNC–Lys material, showing the roughness of the substrates. (**C**) FT–IR spectra of the CNC–Lys (**a**) and CNC (**b**) material, confirming the structural modification of CNC with lysine. (**E**) ^1^H–NMR study of CNC–Lys using D_2_O solvent, showing the attachment of lysine on CNC. (**F**) TGA analysis of the CNC–Lys material, along with its DTA plot, showing the effect of high temperature on the destruction of lysine.

A scanning electron microscopy (SEM) image of CNC–Lys is shown in Figure 1A. It was observed that the surface of CNC–Lys was rough with agglomerated cauliflower–like morphology. The fiber-like structure of CNC Can also clearly be seen in the SEM images. Additionally, the surface morphology of CNC–Lys was also observed by the atomic force microscope (AFM) technique (Figure 1B,D). The imaging was performed in tapping mode using a cantilever tip radius of ~10 nm. The AFM images revealed a highly rough surface with some protrusions coming from the surface. The arithmetic average roughness (Ra) and root mean square average roughness (Rq) of the CNC–Lys were measured to be 6.16 and 8.96 nm, respectively, for a scan area of 10 × 10 μm^2^.

Information about the different chemical functionalities present in CNC–Lys and CNC was obtained using Fourier transform infrared (FT–IR) spectroscopy (Figure 1C). Some common peaks, such as a broad peak at around 3400 cm^−1^, a small peak at 2870 cm^−1^, bands of peaks at 1300–1420 cm^−1^, peaks at 900–1110 cm^−1^, and a small absorption peak at 890 cm^−1^, were observed in the spectra of both CNC and CNC–Lys material. However, some differences in the spectra of CNC and CNC–Lys were also observed. For example, a peak at 1620 cm^−1^ in CNC (Figure 1C–b) was shifted to 1600 cm^−1^ in CNC–Lys (Figure 1C–a). Similarly, two extra small peaks at 1240 and 795 cm^−1^ were observed only in CNC–Lys.

**Figure 2:**
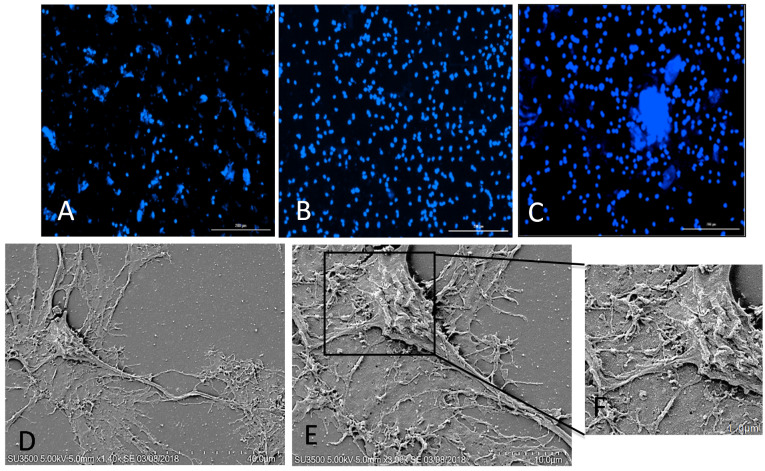
Attachment of rNSC cells to the CNC–, PDL–, and CNC–Lys–coated surfaces and SEM images of differentiated rat NPCs on the CNC–Lys surface. The plated NSCs attached well to the (**A**) CNC, (**B**) PDL, and (**C**) CNC–Lys surfaces, Scale bar: 200 μm. Nuclei were counterstained with DAPI. (**D**) Fully differentiated neurons on the CNC–Lys surface. Scale bar: 40 μm. (**E**) Cell body of neurons with neurites, shown in more detail. Scale bar: 10 μm. (**F**) High magnification of the boxed region, showing details of the surface of the cell body and its interaction with the substratum. Scale bar: 1 μm.

Additionally, proton nuclear magnetic resonance (^1^H–NMR) analysis was performed for CNC–Lys material to observe the attachment of lysine on CNC. The ^1^H–NMR spectrum is shown in Figure 2E, with a plot of relative intensity versus chemical shift. A bunch of proton peaks ranging from 1.0 to 5.0 was observed in the spectrum. One of the peaks at 4.6 ppm was found to be overlapped with the peak of the solvent. Furthermore, the covalent modification of CNC with lysine was also studied by performing TGA of a CNC–Lys sample. The thermal gravimetric analysis (TGA) curve, which was plotted as weight loss (%) versus temperature, and the overlapped differential thermal analysis (DTA) data are presented in Figure 1F. It can be seen from the curves that the mass loss for the CNC–Lys sample occurred in four different steps. An initial mass loss of 3% was observed at 50 °C, but a major mass loss of around 75% was observed in the second step at 250 °C. In the third step, a 10% weight loss of CNC–Lys was seen with a medium peak in the DTA plot. Finally, another 10% weight loss was detected at 810 °C. Moreover, the crystallinity of the CNC–Lys and CNC samples were observed using the X–ray diffraction (XRD) technique, as shown in Figure S1. The five different crystalline peaks (101, 10ī, 021, 002, and 040) were separated with the prominent 002 crystalline peak, in agreement with the literature [60,61,62].

### 2.2. Proliferation and Differentiation of NSCs/NPCs

Culturing E14 rNSCs in Dulbecco’s Modified Eagle’s Medium/nutrient mixture F–12 (DMEM/F–12) serum-free growth medium with fibroblast growth factor (FGF), epidermal growth factor (EGF), and StemPro neural supplement for two weeks resulted in a robust yield of undifferentiated dividing cells (Figure S2). Later, the cells were collected and sub-cultured, which yielded a high number of undifferentiated cells considered as passage 2 rNPCs. The cells in the maintenance medium, where only FGF and other supplements were added while EGF was withdrawn, were plated on the poly–d–lysine (PDL) and CNC–Lys surfaces and differentiated for one, two, and three weeks.

**Figure 3:**
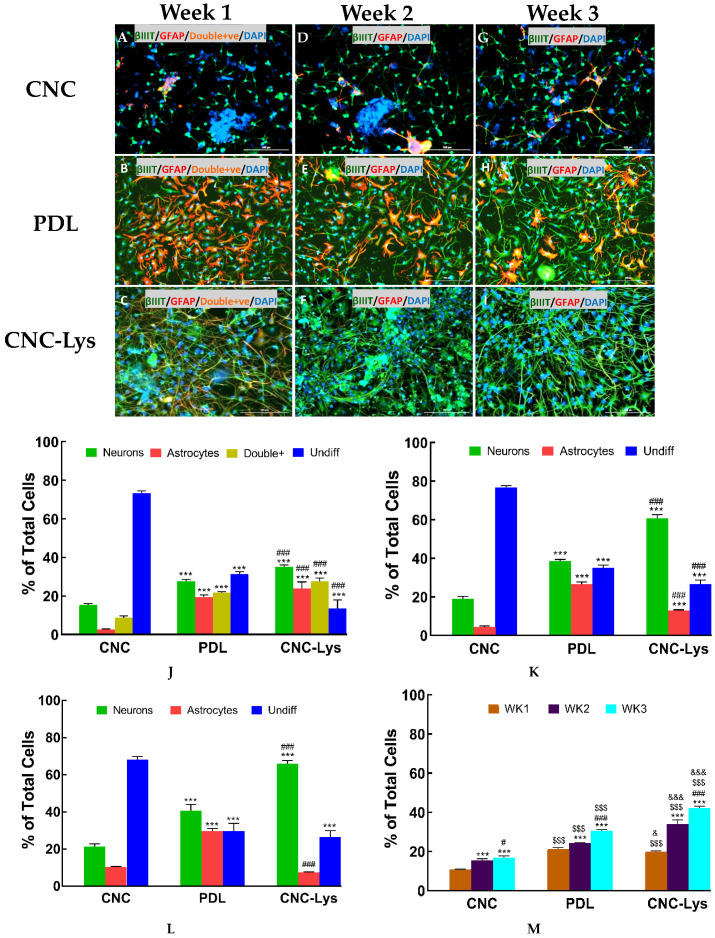
Representative fluorescent images illustrating the promotion of neuronal differentiation by the CNC, PDL, and CNC–Lys surfaces. Neurons and astrocytes are labeled with specific markers for 1 (**A**–**C**), 2 (**D**–**F**), and 3 (**G**–**I**) weeks in culture. Neurons expressing βIII tubulin are green, astrocytes expressing GFAP are red, and the nuclei are counterstained blue by DAPI in all panels. The total population of cells was quantified for each experimental condition with CNC (**A**,**D**,**G**); PDL (**B**,**E**,**H**), and CNC–Lys (**C**,**F**,**I**). The percentage of neurons and astrocytes, as well as undifferentiated or incompletely differentiated (double-positive for both βIII tubulin and GFAP), were quantified at 1 (**J**), 2 (**K**), and 3 (**L**) weeks on the CNC, PDL, and CNC–Lys surfaces. (**M**) We further identified the mature neuronal marker MAPII (see images in Supplemental Figure S2) for 1, 2, and 3 weeks on the CNC, PDL, and CNC–Lys surfaces. (**J**) In week 1, there were significantly more cells differentiated into neurons compared to astrocytes. *** *p* < 0.001 compared to the CNC substratum of the same cell type. ### *p* < 0.001 compared to the PDL substratum of the same cell type. Both the CNC–Lys and PDL surfaces promoted differentiation to a higher degree than the CNC surface. (**K**) By week 2, there was a significantly higher percentage of rNSCs differentiated into neurons on the PDL and CNC surfaces compared to the CNC surface. *** *p* < 0.001 compared to the CNC substratum of the same cell type. ### *p* < 0.001 compared to the PDL substratum of the same cell type. The CNC–Lys surface was excellent for differentiation of cells into neurons. ### *p* < 0.001 compared to the same cell type of the CNC surface in week 2. (**L**) By week 3, there was a further reduction of astrocytes and an increase in neuron cell type on the CNC–Lys (U) surface. *** *p* < 0.001 compared to the CNC substratum of the same cell type. ### *p* < 0.001 compared to the PDL substratum of the same cell type. (**M**) The percentage of mature neurons on the CNC–Lys surface increased from weeks 1 to 3. *** *p* < 0.001 compared to the results of week 1 (WK1) of the same substratum. # *p* < 0.05 and ### *p* < 0.001 compared to the results of week 2 (WK2) of the same substratum. $$$ *p* < 0.001 compared to the CNC substratum at the same time point. & *p* < 0.05 and &&& *p* < 0.001 compared to the PDL substratum at the same time point. Data represented as mean ± SD for *n* = 6. The difference between different conditions and times was tested by a two-way analysis of variance (ANOVA), followed by post-hoc analysis (Tukey’s test) for double-positive cells. Scale bars represent 200 μm.

Our results revealed that the rNPCs attached well to both the PDL and CNC–Lys (Figure 2B,C) surfaces, but very minimally to the CNC surface, possibly due to the lack of a positive charge (Figure 3A). Determination of the total cell count by counting DAPI (4′,6–and diamidino–2–phenylindole, dihydrochloride)–positive cells using Cytation-5 Bioimager showed the presence of 94.23% of total plated cells on the CNC–Lys substratum at seven days in the culture, indicating good adhesion. Meanwhile, 85.31% of DAPI-positive cells were present on the PDL substratum, and only 34.5% of the total plated cells were present on the CNC substratum, indicating that CNC by itself provided very poor attachment to cells, while CNC–Lys provided the maximum attachment. Although the cell numbers declined on the CNC substratum, they remained without significant reduction under the other two conditions in weeks two and three (Table 1). The differentiating rNPCs showed neuronal and astrocytic (Figure S3) markers on the CNC–Lys substratum. The SEM images revealed the detailed morphology of the differentiated neurons from NPCs on the CNC–Lys surface (Figure 2E). The neurons that differentiated on the CNC–Lys surface showed long and branched neuritic processes (Figure 2F).

### 2.3. The Majority of the rNPCs Differentiated into Neurons on the CNC–Lys Surface

**Figure 4:**
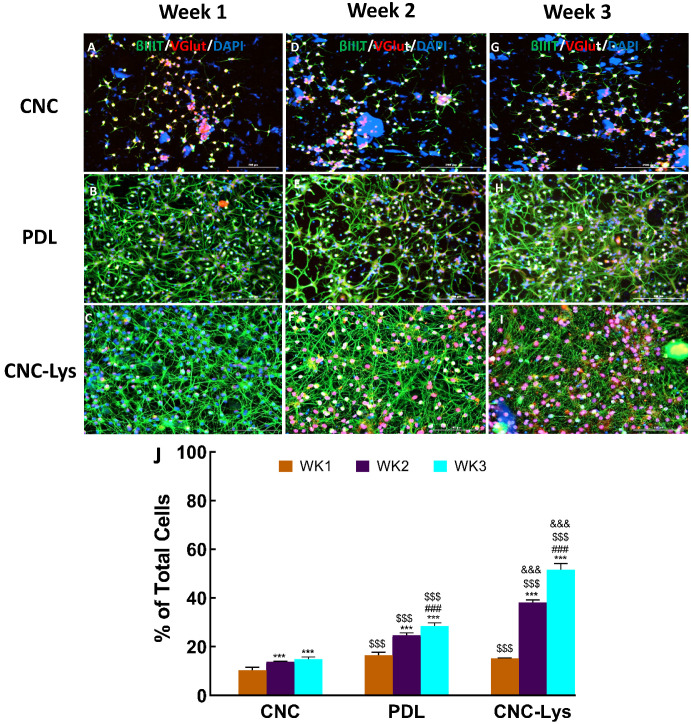
Representative fluorescent images for glutamatergic neurons differentiated on the CNC, PDL, and CNC–Lys surfaces. Neurons were labeled with specific markers for 1 (**A**–**C**), 2 (**D**–**F**), and 3 (**G**–**I**) weeks in culture. Neurons expressing βIII tubulin are green, cells expressing VGlut are red, and all of the nuclei are counterstained blue by DAPI in all panels. The total population of cells was quantified for each experimental condition with CNC (**A**,**D**,**G**), PDL (**B**,**E**,**H**), and CNC–Lys (**C**,**F**,**I**). The percentage of neurons was quantified (**J**) across 3 weeks of duration out of the equal number of total rNSCs plated at the beginning. The glutamatergic neurons increased over time with the most expression on the CNC–Lys surface in week 3. *** *p* < 0.001 compared to the results of week 1 (WK1) of the same substratum. ### *p* < 0.001 compared to the results of week 2 (WK2) of the same substratum. $$$ *p* < 0.001 compared to the CNC substratum at the same time point. &&& *p* < 0.001 compared to the PDL substratum at the same time point. The difference between different conditions and times was tested by a two-way analysis of variance (ANOVA), followed by post-hoc analysis (Tukey’s test). The data represent the mean percentage ± SD for *n* = 4. Scale bars represent 200 μm.

Immunostaining was performed with cultures of rNPCs each week. Our results showed that at seven days, not many cells attached to the CNC-coated surface had differentiated, with 15% of the cells present at one week differentiated into neurons (βIII tubulin-positive) and 3% into astrocytes (glial fibrillary acidic protein (GFAP)-positive, Figure 3A,J) while 72% of the cells remained undifferentiated. On the CNC–Lys surface, 35.13% of the cells present at one week differentiated into neurons, being βIII tubulin-positive (Figure 3C,J) compared to 28% on PDL present at one week (Figure 3B,J), indicating that CNC–Lys surface was more conducive for neuronal differentiation. Furthermore, cells were immunostained for identifying mature neuronal marker protein MAPII. MAPII-positive neurons represented 20% of the total neurons differentiated on CNC–Lys at week 1, comparable to PDL showing 21% MAPII-positive cells (Figure 3M and Figure S3). The unmodified CNC surface had only 11% MAPII-positive cells (Figure 3M and Figure S3) during the same duration of culture. Astrocytic differentiation was found to be similar on the CNC–Lys surface (23.75%) compared to PDL (19.30%) (Figure 3B,C,J). The CNC surface had the least astrocytes in week 1 (3%, Figure 3A). However, 9%, 22%, and 28% of the cells present on CNC, PDL, and CNC–Lys, respectively, co-expressed βIII tubulin and GFAP, suggesting they were not fully differentiated either into neurons or astrocytes (Figure 4J) at this stage. At the end of 14 days in vitro (Figure 3D–F,K), neuronal differentiation on CNC–Lys (60.57%; Figure 3F,K) was significantly higher than that of PDL (39%, Figure 3E,K, *p* < 0.001) and CNC (18.4%, Figure 3D,K, *p* < 0.001), with a much higher mature neuronal count (MAPII-positive, 34%, Figure 3M and Figure S3) as well. The cells maintained in culture for 21 days resulted in 66%, 41%, and 21% of the cells differentiating into neurons on the CNC–Lys, PDL, and CNC surfaces (Figure 3G–I,L), respectively, with 42% of the cells being mature neurons on the CNC–Lys surface (Figure 3M and Figure S3).

**Figure 5:**
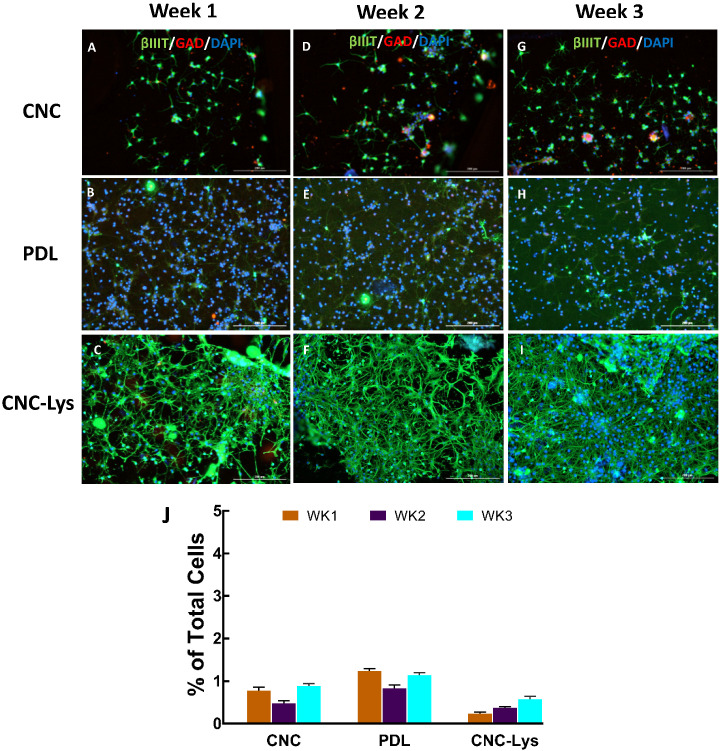
Representative fluorescent images for GABAergic neurons differentiated on the CNC, PDL, and CNC–Lys surfaces. Neurons were labeled with specific markers for 1 (**A**–**C**), 2 (**D**–**F**), and 3 (**G**–**I**) weeks in culture. Neurons expressing βIII tubulin are green, cells expressing GAD are red, and all of the nuclei are counterstained blue by DAPI in all panels. The total population of cells was quantified for each experimental condition with CNC (**A**,**D**,**G**), PDL (**B**,**E**,**H**), and CNC–Lys (**C**,**F**,**I**). The percentage of neurons was quantified across 3 weeks on the CNC, PDL, and CNC–Lys surfaces (**J**). GAD was expressed very minimally in neurons on the CNC (**A**–**C**) and PDL surfaces (**D**–**F**), and almost none on the CNC–Lys surface (**G**–**I**). Data represent the mean percentage ± SD for *n* = 4. Scale bars represent 200 μm.

**Figure 6:**
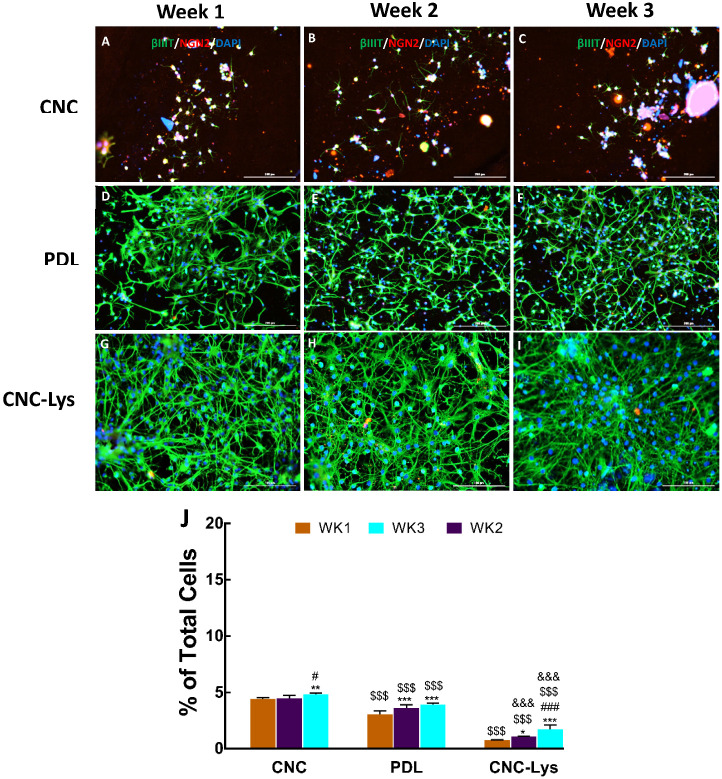
Representative fluorescent images for motor neurons differentiated on the CNC, PDL, and CNC–Lys surfaces. Neurons were labeled with specific markers for 1 (**A**–**C**), 2 (**D**–**F**), and 3 (**G**–**I**) weeks in culture. Neurons expressing βIII tubulin are green, cells expressing NGN2 are red, and all the nuclei are counterstained blue by DAPI in all panels. The total population of cells was quantified for each experimental condition with CNC (**A**,**D**,**G**), PDL (**B**,**E**,**H**), and CNC–Lys (**C**,**F**,**I**). The percentage of neurons was quantified across 3 weeks on the CNC, PDL, and CNC–Lys surfaces (**J**). Motor neuron marker NGN2 was minimally expressed on the CNC (**A**–**C**) and PDL surfaces (**D**–**F**), and almost none on the CNC–Lys surface (**G**–**I**). * *p* < 0.05, ** *p* < 0.01, *** *p* < 0.001 compared to the results of week 1 (WK1) of the same substratum. # *p* < 0.05 and ### *p* < 0.001 compared to the results of week 2 (WK2) of the same substratum. $$$ *p* < 0.001 compared to the CNC substratum at the same time point. &&& *p* < 0.001 compared to the PDL substratum at the same time point. The difference between different conditions and times was tested by a two-way analysis of variance (ANOVA), followed by post-hoc analysis (Tukey’s test). Data represent the mean percentage ± SD for *n* = 4. Scale bars represent 200 μm.

The expression of vesicular glutamate transporters 1 and/or 2 (VGLUT1/2), gamma-aminobutyric acid (GABA) (glutamic acid decarboxylase–65 (GAD–65)), and neurogenin2 (NGN2) (spinal motor neuron marker) in differentiated neurons was examined to ascertain the degree of differentiation of cells into a particular neuronal lineage (Figure 4, Figure 5 and Figure 6). The expression of a glutamatergic marker protein in differentiated neurons increased over time, reaching 51.6% on the CNC–Lys surface by the end of the week 3 culture (Figure 4I,J), significantly higher than that observed in the cells on the PDL (Figure 4H,J) and CNC (Figure 4G,J) surfaces. The GAD expression was low but significant in neurons differentiated on the CNC (0.9%, Figure 5A,D,G,J) and PDL (1.1%, Figure 5B,E,H,J) surfaces, whereas it was almost undetectable on the CNC–Lys surface (0.6%, Figure 5C,F,I,J). Neurons expressing the marker for the potential motor neuron phenotype were observed on both the CNC and PDL surfaces at 4.8% (Figure 6C,J) and 3.9% (Figure 6F,J) by week 3, respectively. The neurons on CNC–Lys did not express any significant level of NGN2 across all time durations (Figure 6G–J).

### 2.4. Electrophysiological Activities of Neurons Differentiated on CNC–Lys

**Figure 7:**
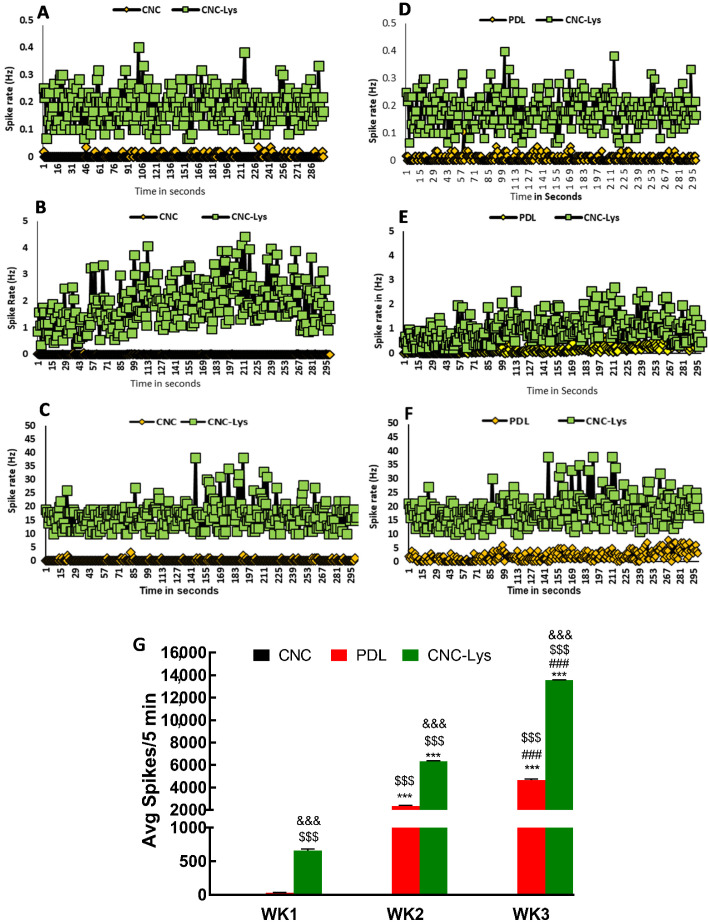
Time-dependent increase in spontaneous electrical signals from cells differentiated on the CNC, PDL, and CNC–Lys surfaces across 3 weeks. (**A**–**C**) Representation of the population spikes (5 min) generated at each second by neurons differentiated on the CNC–Lys or CNC (control) surfaces in MEA dishes for weeks 1 (**A**), 2 (**B**), and 3 (**C**) in vitro. (**D**–**F**) Representation of the population spikes (5 min) generated at each second by neurons differentiated on the CNC–Lys or PDL (control) surfaces in MEA dishes for weeks 1 (**D**), 2 (**E**), and 3 (**F**) in vitro. The cumulative spike rates (Hz) from the neurons in (CNC, PDL, and CNC–Lys) the entire dish across 300 sec is illustrated in (**G**). The spontaneous activities of the neurons on the CNC surface were much lower than those on the PDL and CNC–Lys surfaces throughout the 3 weeks. The spontaneous activity increased dramatically over time on the PDL and CNC–Lys surfaces. *** *p* < 0.001 compared to the results of week 1 (WK1) of the same substratum. ### *p* < 0.001 compared to the results of week 2 (WK2) of the same substratum. $$$ *p* < 0.001 compared to the CNC substratum at the same time point. &&& *p* < 0.001 compared to the PDL substratum at the same time point. The difference between different conditions and time was tested by a two-way analysis of variance (ANOVA) followed by post-hoc analysis (Tukey’s test). Data represent mean percentage ± SD for *n* = 3.

The cells that were maintained in differentiating medium and cultured on CNC–, PDL–, and CNC–Lys-coated MEA dishes for 300 s by weeks 1, 2, and 3 (Figure 7) were used for recording the electrical field potential. The cells exhibited extracellular local field potentials (spontaneous activity) captured by the MEA amplifier and were duly recorded (Figure S4). After one week of differentiation, the cells cultured on the CNC–Lys material exhibited spontaneous firing at 0.25 spike/sec/neuron (Figure 7D), and by the end of the second week, it increased to 1 spike/sec/neuron (Figure 7E) associated with occasional bursts from the neuronal networks. Additionally, we observed that the cells differentiated on CNC did not exhibit any activity in weeks 1 or 2 (Figure 7A,B). It was observed that although cells differentiated to appear morphologically as neurons were present on the detection field of most of the electrodes, not all cells were found to be active or to fire during recording. However, by the end of the third week in culture on CNC–Lys, the network of cells with neuronal morphology that could be visualized under the microscope while recording showed a high level of spontaneous activity at 200 spike/sec/cell (Figure 7F,G). Even by the end of week 3, cells on CNC did not exhibit any detectable firing (Figure 7C,G). The level of network activity indicated by an average number of spikes increased with the duration of culture (Figure 7G), consistent with the increased percentage of glutamatergic neuronal population in the culture over time, as demonstrated in the immunocytochemical results (Figure 4I,J). Cells that appeared to be neurons on the CNC and PDL surfaces showed much less activity compared to CNC–Lys (Figure 7G). The spontaneous activities of the cells cultured on these surfaces seem to be consistent with the cell attachment and patterns of the neuronal differentiation revealed by cell count and immunostaining (Figure 3M and Figure 4J). Since the cells were plated and cultured on MEA dishes similar to how they were plated and cultured on dishes in other conditions, we assume that the differentiation of cells into neurons on the MEA would be very similar to that of the differentiation pattern of cells on the dishes.

## 3. Discussion

We newly synthesized CNC–Lys material and fully characterized its morphology and chemical bonding using the SEM, AFM, FT–IR, TGA, and ^1^H–NMR spectroscopic techniques to demonstrate the successful attachment of lysine with CNC. Our cell culture data indicated that CNC–Lys is an excellent surface for neuronal differentiation from rNSCs in respect to its surface property, cell adhesion, cytotoxicity, and promotion of rNSC differentiation toward neuronal lineage.

SEM imaging (Figure 1A) revealed a cauliflower-shaped structure observed on the surface of the material due to the attachment of lysine to the CNC, indicating the successful attachment of lysine molecules to the CNC. The fibrous structure of CNC–Lys observed in the SEM images confirmed that our material is suitable for mimicking the ECM environment for cell differentiation. The roughness of the material is crucial, as it determines the adhesive behavior of the material on the substrate during the cell culture experiment [63]. As shown in the AFM images (Figure 1B,D), some fibrous protrusions were observed on the surface of CNC–Lys, which were responsible for the increased roughness of the sample. The roughness of CNC–Lys successfully facilitated the adhesion of cells on the substrate during the cell culture experiment.

The FT–IR study further verified the attachment of lysine on the CNC surface. A broad peak at around 3400 cm^−1^ and a small peak at 2860 cm^−1^, seen in both the CNC and CNC–Lys spectra (Figure 1C), were due to O-H and C-H bond stretching, respectively. However, in the CNC spectrum (Figure 1C–a,b), the peak due to N-H stretching coming from the lysine structure was not distinguishable because of the overlapping of the peak with the O-H stretching peak. The O-H bending mode observed in CNC at 1620 cm^−1^ was shifted to 1600 cm^−1^ in CNC–Lys, which was broad and of high intensity due to the overlapping of N-H bending, especially from the primary amine present in lysine. Bands at 1300–1420 cm^−1^, present in both spectra, were due to the deformation vibration of the C-H group present in CNC and lysine. Sim ilarly, the peaks at 900–1110 cm^−1^ were assigned to the –C–O– group of the secondary alcohol and ether functions present in the CNC backbone. A small absorption peak found at 890 cm^−1^ was the characteristic of β-glycosidic linkage between glucose units of CNC [64]. The two extra small peaks at 1240 and 795 cm^−1^, belonging to C-N stretching and –NH_2_ wagging, respectively, as shown in the CNC–Lys spectrum (Figure 1C–a), further support the attachment of lysine on the CNC surface [65].

The modification of CNC with lysine was also studied by the chemical shift observed in the ^1^H–NMR spectrum (Figure 1E). The chemical shift, ranging from 2.9 to 4.4 ppm, was due to the presence of proton on the CNC backbone. Additionally, some proton peaks originating from lysine overlapped with the peaks from the CNC backbone. The proton peaks ranging from 1.0 to 1.8 ppm were also from lysine. The amine peak present in the tail of lysine was observed at 1.7 ppm, overlapped with the methylene proton peak of lysine, as shown in Figure 1E. After analyzing the peaks in the NMR spectrum, it was confirmed that CNC–Lys was perfectly constructed during synthesis. By analyzing the weight loss of the sample with respect to temperature, the constituents of the materials could be predicted. In the TGA plot (Figure 1F), a small drop of weight observed at the beginning was ascribed to be the evaporation of adsorbed water molecules present in the sample. The pronounced weight loss that occurred in the second step at 250 °C was mainly due to the degradation of CNC [38,66,67]. The lysine was thermally stable up to 250 °C, but started to degrade beyond this temperature. The lysine moiety of the CNC–Lys sample was completely degraded in the third step (at 430 °C) with a weight loss of 10%. In the last step, complete evaporation of char formed by the decomposition of CNC–Lys was accompanied by a 10% weight loss at 810 °C. The crystallinity of nanocellulose is one of the important properties for its accessibility; however, it can be affected by chemical modifications [60]. As shown in Figure S1, a decrease in the intensity of each crystalline peak of nanocellulose was observed after the chemical modification. For example, the intensity of the 002 crystalline peak at 23.4° was less for modified nanocellulose (CNC–Lys) (Figure S1, purple spectrum) compared to that of pure CNC (Figure S1, red spectrum). However, the above results of the NSC differentiation show that the retained crystallinity of the modified nanocellulose (CNC–Lys), even after the chemical modification, was good enough to mimic the microenvironment of EMC and enhance the differentiation of NSCs into neurons.

**Figure 8:**
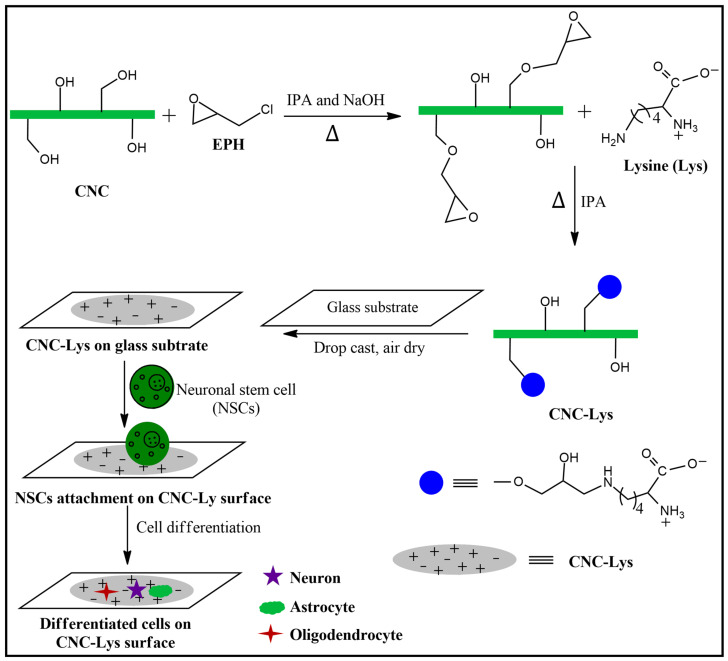
Schematic representation of the synthesis of nanocellulose coupled with lysine molecules (CNC–Lys) and its use for NSCs = differentiation.

Cell adhesion is a major aspect in an in vitro cell culture that determines the final fate of cells. The attachment of cells to surface materials involves the presence of a positive charge, roughness, and stiffness of the material constituting the substratum. Courtenay et al. suggested that cellulose material requires the addition of positive molecules or modification using chemicals to enhance the attachment of cells [68]. Our work showed that CNC coupled with lysine molecules provides better attachment of cells by the scheme illustrated in Figure 2C and Figure 8, probably due to both the roughness and presence of positive charges. The roughness of the material plays a crucial role in the attachment of cells to the surface [63]. The AFM analysis results (Figure 1C,D) revealed that the CNC–Lys surface was rough enough to allow attachment of cells for its proliferation and differentiation. Furthermore, material stiffness has also been a factor in the attachment of cells to biological materials. The usual stiffness of CNC is between 110 and 220 Pa [69]. Earlier studies have confirmed that the minimum stiffness of cellulose needs to be higher than 100 Pa for NSCs to grow and differentiate [70]. In our work, the cells attached and differentiated well on the CNC–Lys surface, confirming that the roughness and stiffness of the cellulose orient stem cells toward differentiation.

Cell affinity for a biomaterial is governed by cell–matrix interactions, usually resulting from specific recognition of cell surface adhesion receptors such as integrin by the extracellular matrix. Naturally occurring cellulose does not have integrin-binding sites to facilitate cell attachment [46,71,72]. Therefore, modification of the surface is necessary to enhance surface attachment and cell–scaffold interactions for cells to attach and proceed to differentiate [73,74]. The fibrous structure of CNC observed during SEM imaging (Figure 1A), along with coupled lysine molecules adding the positive charges to the CNC material, revealed by the FT–IR and NMR analysis indeed promoted cell–ECM interactions.

The immunofluorescence data revealed that 35% and 23.75% of total plated rNSCs expressed βIII tubulin and GFAP, respectively, after one week in culture on CNC–Lys (Figure 3C,J), and MAP2–positive cells with mature neuronal lineage reached 20% (Figure 3M and Figure S3). This is in agreement with the earlier reports by Stabenfeldt et al., who showed that primary murine neurospheres cultured on methylcellulose scaffolds coupled with laminin enhance rNSC survival and maturation and promote neuronal differentiation [75]. In contrast, cells cultured on CNC revealed a different pattern with regard to the differentiated cell counts. The week 1 results revealed only 15% of neuronal differentiation and 3% of the astrocytic lineage of the total cells plated. The first week’s results suggest that stem cells differentiated well on the CNC–Lys surface and showed more mature neurons compared to the CNC surface, which could be a result of more cells being attached to the CNC–Lys substratum than the CNC substratum (Table 1). In the week 2 cultures, we did not observe any cells co–expressing both βIII tubulin and GFAP, but 60.58% of the differentiated cells were βIII tubulin–positive on the CNC–Lys surface (Figure 3F,K). Additionally, 34% of the differentiated cells expressed mature neuron marker MAP2 (Figure 3M and Figure S3). The rate of neuronal differentiation was higher in week 2 compared to week 1. Then, the enhancement of differentiation slowed down during week 3, suggested by only a 6% increase in the number of neurons on CNC–Lys over week 2 differentiation (Figure 3I,L). On the CNC surface, the neuronal cell count could only reach a maximum of 21% by the end of week 3, much lower than that on the PDL and CNC–Lys surfaces (Figure 3G,L), further proving that lysine molecules attaching to CNC does aid in the differentiation of rNSCs and that the several lysines with positive charges present in the PDL might have contributed to the successful neuron differentiation observed on the PDL surface as well. Neurons differentiated from stem cells exhibited dense neurite processes on the CNC–Lys surface (Figure 2E and Figure S3), consistent with the report that cellulose favored neurite outgrowth of neuroblastoma cells [76].

Glial fibrillary acidic protein (GFAP) is an astrocytic marker protein expressed by astrocytes. Surface stiffness likely plays a role in the expression of GFAP. The cells, once attached to the surface, may increase the synthesis of the GFAP protein in response to increased surface stiffness. Fuchs and Weber in 1994 reported that the upregulation of intermediate filament GFAP is due to the adhered cells sensing the strength of the surface. Later work by Min et al. in 2015 suggested that GFAP expression increased due to astrocytes sensing the stiffness of the cellulose acetate nanofiber [77]. The most interesting finding in our current work is that NSC differentiation toward astrocytic lineage decreased considerably by the end of week 3 (Figure 3I,L)—the astrocyte count was 28% in week 1, and by week 3, it dropped to 7.4% on the CNC–Lys surface. In contrast, the percentage of astrocytes on the PDL surface remained unchanged (Figure 3B,E,H,L). These results reveal that the nanocellulose material promotes neuronal rather than astrocytic differentiation (Figure 3L). Moon et al. in 2011 determined that CNC (BGB ultra cellulose nanocrystals No. 3912.90) exhibits a tensile strength of 7.5–7.7 GPa, elastic modulus in the axial direction of 110–220 GPa, and elastic modulus in the transverse direction of 10–50 GPa [69]. The same material was used in this current study, with less stiffness than that of cellulose acetate nanofiber used by Min et al. [77]. It is possible that the CNC material we used here lacked the required stiffness or elasticity to promote GFAP expression, or the poor attachment of cells onto the CNC surface could have also contributed to fewer cells exhibiting GFAP staining.

Immunostaining with specific marker proteins is an established method for identifying different cell types, such as glutamate for excitatory neurons and GABA for inhibitory neurons and NGN2 for motor neurons. Therefore, we chose to evaluate the endogenous expression of proteins related to glutamate, GABA, and motor neurons using specific antibodies. The major excitatory neurotransmitter glutamate acts both as an amino acid and a neurotransmitter. Glutamate receptors are found throughout the brain and spinal cord in neurons and glia. Glutamate has a large array of normal physiological functions, including glutamate synapse control or the modulation of neuronal excitability [78]. VGLUT1 and VGLUT2 are the most abundant isoforms expressed in glutamatergic neurons in the cortex, hippocampus, thalamus, and cerebellar cortex [79]. Motor neurons and GABA neurons are also widely present in the CNS. GABAergic neurons are the major inhibitory neurons in the CNS [80]. L–Glutamic acid decarboxylase (GAD) is the major enzyme converting glutamate into GABA. NGN2 plays a unique and critical role in determining motor neuron cell–type identity [81]. Our work revealed that most of the neurons matured and they were mostly glutamatergic by the end of week 3 (Figure 4I,J), indicated by a marker for endogenous expression of VGLUT. Notably, the rNSCs differentiated into neurons on CNC–Lys did not express any significant levels of GAD (Figure 5C,F,I,J), suggesting that CNC–Lys promoted the differentiation of glutamatergic neurons rather than GABAergic neurons. Similarly, the neurons differentiated on the CNC (Figure 5A,D,G,J) and PDL (Figure 5B,E,H,J) surfaces showed much lower expression of GAD compared to VGLUT. Only a few cells expressed NGN2 on CNC–Lys (Figure 6C,F,I,J), and slightly more cells did so on the CNC (Figure 6A,D,G,J) and PDL (Figure 6B,E,H,J) surfaces in all time points in the culture. This result indicates that rNSCs differentiated more toward the glutamatergic lineage rather than the GABAergic or motor neuron cell types, which is probably due to the surface characteristics that helped neurons to orient toward the glutamatergic type. The percentage of neurons that were glutamatergic on CNC–Lys was significantly higher compared to on CNC. Interestingly, those neurons on CNC produced the most motor neuron phenotype, reaching a maximum of 4.8% by the end of week 3 (Figure 6G). It remains to be seen whether the change in surface properties/characteristics would lead to the shifting of rNSC differentiation into specific type(s) of neurons. Apart from neurons differentiating across different time periods, we also observed that the surface played a role in promoting rNSCs to differentiate into neurons. Among all three surfaces we used, CNC–Lys promoted higher neuronal differentiation and maturation of neurons, and differentiated into the glutamatergic neuronal phenotype.

To characterize the electrophysiological activity of the neural stem cells undergoing differentiation in vitro longitudinally, non-invasive studies play a crucial role in the study of neuronal dynamics. This is a highly complex process that depends upon a large number of interrelated dynamic factors regulating neuronal excitability [82]. Van Pelt et al. suggested that the study of neuronal networks in vitro is conceivable, because the functional characteristics of ex vivo neuronal networks are similar to those observed in vivo in terms of connectivity, the inhibition/excitation ratio, and electrophysiological and electrical stimuli [83].

In the current study, we evaluated the spontaneous activity of NSC-derived NPCs undergoing differentiation on CNC–, PDL–, and CNC–Lys–coated MEA recording dishes. This is the first report of spontaneous electrical activity recorded from neural stem cells differentiating on a CNC–Lys surface for three weeks. Spontaneous activity is a common characteristic of developing neuronal networks both in vivo and in vitro, which is believed to play an important role in network development [83,84,85,86,87]. The recurrent synchronous electrophysiological activity in the developing neurons in the nervous system is similar to that exhibited by neuronal cultures in vitro [88,89]. Our immunocytochemical results revealed differentiated neuronal populations (Figure 4C,F,I,L) that coincide with the spontaneous electrical activities from the electrophysiological recordings (population spikes, Figure 7A–C). Earlier work by Czarnecki et al. in 2012 reported spontaneous burst activity in a primary cortical network in vitro [90]. Most of the neural cultures lead toward the formation or differentiation of a heterogeneous mixture of neurons unless generations of pure neuronal cultures such as GABAergic and glutamatergic neurons are produced [91]. Here, only recorded data from electrodes with distinct long-term stable electrophysiological activity were analyzed and included in the statistical analysis. Interestingly, it was observed that not all of the cells exhibited a regular or synchronous pattern of activity. The electrical activity recorded increased (Figure 7G) across the three weeks, concurrent with increased percentages of cells expressing neuronal markers from weeks one to three. Earlier studies have reported that MEAs also help in the identification of neuronal subtypes such as glutamatergic and GABAergic derived from mouse embryonic stem cells [92,93] by identifying their excitatory or inhibitory influence. The increased number of glutamatergic neurons or enhanced expression of vesicular glutamate transporters 1 and 2 in the cultured neurons over time may contribute to the increase in the electrical activity exhibited by the differentiated neurons on the MEA dishes, more so on the CNC–Lys surface, since relatively more cells differentiated into neurons on this surface. Earlier work by Ito et al. using MEAs observed an increase in electrical activity in cultured rat cortical neurons forming a network over three weeks in a culture similar to our study [52].

The lower number of astrocytes on the CNC–Lys surface also supports that with a longer duration of culture in vitro on the CNC–Lys surface, stem cells differentiate more into neurons that may facilitate network formation (Figure S3 and Figure 3I). The average of all the spikes (5 min/dish, three dishes per condition) recorded from the neurons on the CNC–Lys surface was approximately 13,500 spikes compared to 4653 spikes on PDL and a mere seven spikes on the CNC-coated surface when recorded at the end of week 3 (Figure 7G), consistent with the differentiation pattern of cell types on different surfaces. The lysine-bound CNC–Lys did indeed play a crucial role in cell attachment and differentiation with profuse network formation (Figure 4I), leading to increased electrical activity from the electrophysiology data (Figure 3I, Figure 7C, and Figure S4). Since PDL also provides lysine to the substratum, it appears that the combination of cellulose roughness and the positive charges from lysine together could have contributed to the increased neuronal differentiation we observed on the CNC–Lys culture surface. Earlier work by Muramoto et al. established that an increased number of synapses could increase the frequency of spontaneous activity [94]. Therefore, the neuronal network formed on the CNC–Lys surface (Figure S4) might have led to enhanced synchronous activity.

## 4. Materials and Methods

### 4.1. Reagents and Chemicals

All of the chemicals were analytical grade reagents and used as received, unless otherwise specified. BGB Ultra cellulose nanocrystal (CNC) (HS no. 3912.90) was obtained from Blue Goose Biorefineries Inc. (Saskatoon, SK, Canada). L-lysine and PDL (Cat no. P6407) were purchased from Sigma-Aldrich (St. Louis, MO, USA). Epichlorohydrin (EPH) was obtained from Acros Organics (Pittsburgh, PA, USA). Sodium hydroxide (NaOH) and isopropanol (IPA) were obtained from Fisher Scientific (Pittsburgh, PA, USA). Deionized (DI) water was used to prepare all solutions. Ultra-high purity nitrogen (N_2_) was obtained from NLR Welding Company, (North Little Rock, AR, USA). Dulbecco’s DPBS with and without Ca^2+^ and Mg^2+^, 4% paraformaldehyde, primary antibody to GFAP, and DAPI were purchased from Sigma-Aldrich. Cell Start, DMEM/F-12 medium, StemPro neural supplement, neurobasal medium, TrypLE, B−27, glutamine, glutamax, and Knockout serum replacement (KSR^®^) were purchased from Gibco (Carlsbad, CA, USA). EGF and FGF−2 were purchased from Alomone (Jerusalem, Israel) and noggin from Peprotech (PeproTech, NJ, USA). Mouse anti-β-tubulin-III was purchased from Developmental Studies Hybridoma Bank (DSHB) (Hybridoma product E7 was deposited to the DSHB by Klymkowsky, M.). Primary antibodies to MAP2, VGLUT1/2, and NGN2 were from Cell Signaling (Danvers, MA, USA). The primary antibody to GAD65 Hybridoma Product was deposited to the DSHB by Gottlieb, D.I.

### 4.2. Preparation of the Nanocellulose Surface (CNC–Lys)

The procedure for the synthesis of the CNC–Lys substrate was adopted from previous literature with minor modifications [95,96]. First, 1 g (6.17 mmol) of CNC (12.5 mL of 8% *w*/*w*) was taken in a 100 mL round bottom flask. Then, 30 mL of IPA was added and stirred under N_2_ gas. After 30 min, 0.247 g (6.17 mmol) of NaOH was added and stirred at 40 °C for another 30 min. Then, 1.45 mL (18.49 mmol) of EPH was added and stirred for 4 h. After 4 h, 0.82 mL (6.17 mmol) of L–lysine was added and stirred overnight at 40 °C under N_2_ gas. Since EPH also acts as a cross–linking agent, some of the added EPH might have cross-linked cellulose units [97]. The use of excess EPH molecules ensured that EPH was reacted to cellulose primarily via the –C6–OH group to produce epoxy cellulose ether. Then, an amination reaction occurred between the amine group of L–lysine and epoxy cellulose ether to produce CNC–Lys [95,96]. Finally, the resultant product obtained was washed with excess IPA and DI water to remove unreacted reagents and dried in a vacuum oven. A schematic representation of the synthesis of CNC–Lys is shown in Figure 8.

### 4.3. Characterization of CNC–Lys

The surface morphology of CNC–Lys was studied using JEOL (JSM 7000F Joel, Peabody, MA, USA) SEM. The imaging was performed at a 5 kV applied potential and a 64 μA applied current. The roughness of CNC–Lys was measured using the Bruker dimension icon AFM technique (Billerica, MA, USA). The thin film of CNC–Lys was deposited on a 1 × 1 cm^2^ glass substrate by a spin coating method. AFM imaging of the thin film was performed using the following scan parameters: Scan size (10.0 μm), aspect ratio (1.00), scan rate (0.250 Hz), and samples/line (256). FT–IR analysis was completed using a Nicolet 6700 Thermo Scientific FT–IR spectrometer (Waltham, MA, USA) equipped with a DLaTGS detector and a XT–KBr beam splitter. KBr pellets were made for the FT–IR analysis and the spectra were recorded in the range of 400–4000 cm^−1^. The ^1^H-NMR spectrum of CNC–Lys was obtained using a Jeol 400 MHz NMR instrument (Peabody, MA, USA). The spectrum was collected at 25 °C, and the chemical shifts are in ppm (δ) relative to tetramethylsilane (TMS) as an external standard, unless otherwise stated. A pinch of the sample was taken in an NMR tube and D_2_O solvent was used to analyze the ^1^H-NMR. TGA was performed with a Shimadzu DTG-50 thermal analyzer (Columbia, MD, USA) under an N_2_ atmosphere. CNC–Lys sample weighing 3–4 mg was heated from 25 to 850 °C at a heating rate of 5 °C/min. The samples were tested in triplicates to check the accuracy of the data. The crystallinity of the CNC–Lys and pure CNC samples were studied using the XRD technique, performed in the Bruker D8 Discover instrument (Billerica, MA, USA).

### 4.4. Culture of Rat Neural Stem Cells (rNSCs) and Their Propagation and Passage

Rat embryonic (E14) brains were purchased from BrainBits, LLC (Cat#SKU: SDCCX, Springfield, IL, USA), and the tissue was dissociated according to the vendor’s protocol. The cells were then plated in a T-75 cm^2^ flask pre-coated with CELLStart working solution, as per the manufacturer’s instructions, and placed at 37 °C in a humidified atmosphere of 5% CO_2_ for 1 h. The cells were plated at a density of 1 million per T-75 cm^2^ flask. The cells were maintained undifferentiated in StemPro NSC serum-free medium (Grand Island, NY, USA) with DMEM/F12 medium supplemented with 2% StemPro neural supplement, glutamine (100 mM), glutamax (100 mM), basic FGF, and EGF at 20 ng/mL each. The culture medium was changed every three days and observation was made every day until the cells attained 70–90% confluency. The cells were released from the surface using pre-warmed TrypLE treatment for 30 s at 37 °C and were mechanically dissociated to achieve a single cell suspension. The number of viable cells was counted by a trypan blue exclusion assay in a hemocytometer (Corning, Steuben County, NY, USA). The cells were re-plated in new culture flasks at a density of 1 × 10^6^ cells/mL with fresh culture medium added to with 50 ng/mL of noggin (Peprotech), BMP (bone morphogenetic protein) receptor inhibitors, to aid the expansion of stem cells and inhibit astrocytic differentiation [98]. The cells were collected when they were 75–90% confluent, as described above, and considered as “Passage 2”, which were used for all of our experiments to minimize experimental variation.

### 4.5. Preparation of Culture Surfaces

For plating the cells, 24–well glass-bottomed plates were used. The CNC–Lys suspension was prepared at a concentration of 8% *w/v* in 100% ethanol. Prior to coating the glass surface with the CNC–Lys dispersion, the CNC–Lys suspension was subjected to sonication for 3 min to disperse the cellulose clumps. Then, 100 µL of the CNC–Lys suspension was added to each well and allowed to air dry, after which they were exposed to ultraviolet light overnight to kill any bacteria harbored on the surfaces. Using the same procedure, a plain CNC–coated substrate was prepared and used as a comparator. Similarly, a PDL-coated surface was prepared and used as another comparator. PDL is a polymer of lysine, which has been routinely used in cell cultures [99]. The PDL-coated surface was prepared by incubating the plates with 80 µg/mL of the PDL solution in borate buffer overnight at room temperature. The next day, PDL-coated wells were washed with sterile filtered nanopure water to remove unbound PDL and allowed to air dry in the hood.

### 4.6. Differentiation of Neural Progenitor Cells (NPCs)

To determine the phenotypes of differentiated cells and the rate of differentiation of rNSCs on the CNC, PDL, and CNC–Lys surfaces, the proportion of each cell type was measured. Briefly, ~10,000 cells/well of NPCs were plated onto individual CNC-, PDL-, and CNC–Lys-coated 24–well plates. The cells were maintained in maintenance medium (complete StemPro^®^ medium, Grand Island, NY, USA) for one day before being switched to differentiation medium. The differentiation medium consisted of neurobasal medium, 2% B-27, 100 mM glutamine, 100 mM glutamax, and 1% KSR^®^. Cultures were maintained at 37 °C with 5% CO_2_ for one, two, and three weeks for most of the cells to complete neuronal differentiation. At the end of the culture, the cells were fixed and processed for immunocytochemical identification of neurons and astrocytes using appropriate antibodies.

### 4.7. Scanning Electron Microscopy (SEM) for Morphological Examination of Differentiated rNSCs on the CNC–Lys Surface

Rat NSCs differentiated on the CNC–Lys surface were fixed overnight in 2.5% glutaraldehyde prepared in 1.5% paraformaldehyde in phosphate-buffered saline (PBS) (pH 7.2). The cells were washed with PBS thrice each time for 3 min, and then post-fixed in 2% osmium tetroxide in DI water for 15 min. The cells were rinsed three times with DI water for 5 min each, then dehydrated in a graded series of ethanol solutions, followed by complete dehydration to critical point dry (CPD). The following day, the samples were sputter-coated (EMS 150T ES) with a thin layer (7 nm) of gold to allow surface conductivity. Then, SEM imaging was performed using a HITACHI SU3500 (Santa Clara, CA, USA) equipped with a cold-field emission gun at an acceleration voltage of 5 kV.

### 4.8. Immunostaining and Imaging

Routine immunocytochemistry was performed as follows: Cultures were fixed in 4% paraformaldehyde for 12 min. After washing three times (3 min each) with PBS, the non-specific binding of the antibodies was blocked using tween-20 PBS (PBST) blocking buffer (with Glycine and Bovine Serum Albumin). The cells were then incubated overnight at 4 °C with a primary antibody solution prepared in PBS with 3% goat serum and 0.5% Triton X-100 for permeabilization. The primary antibodies used were mouse anti-β-tubulin-III for neurons and mouse anti-GAD65 for GABAergic neurons (both from DSHB), as well as rabbit anti-GFAP antibody (Sigma) for astrocytes, rabbit anti-MAPII antibody for mature neurons, rabbit anti-VGLUT1/2 for glutamatergic neurons, and rabbit anti- NGN2 for motor neurons (all from Cell Signaling). The next day, after washing out the unbound primary antibodies with PBS three times, appropriate secondary antibodies were added—AlexaFluor488 goat-anti-mouse and AlexaFluor594 goat-anti-rabbit (Invitrogen). Finally, the cells were stained with DAPI. The appropriately stained cells were visualized by fluorescence microscopy using BioTekCytation-5 Bioimager/Plate Reader (Winooski, VT, USA). Gen−5 analysis software (3.08 BioTek, Winooski, VT, USA) was used to count and quantify the different cell populations, unbiased, based on fluorescence.

### 4.9. Plating of Cells in CNC-, PDL-, and CNC–Lys-Coated MEA Dishes

A commercially available MEA system (Multi-Channel Systems GmbH, Reutlingen, Germany) was used to detect the electrical activity of the differentiated cells weekly across three weeks. The MEAs were sterilized according to the manufacturer’s recommendation. Each dish used was coated with 100 µL of CNC, CNC–Lys, or PDL as mentioned before. The rNSCs (passage 2) were plated on CNC–, CNC–Lys–, and PDL–coated MEA dishes containing complete StemPro medium and maintained in an incubator at 37 °C with 5% CO_2_. On the following day, the medium was changed to a neuronal differentiation medium with KSR^®^. Two-thirds of the medium was changed every third day to allow cells to differentiate into neurons and develop electrophysiological activity, and the cultures were maintained for three weeks.

### 4.10. Recording from the MEA Dishes

MEAs for in vitro applications were used. The MEA1060 head stage is composed of 60 titanium nitride microelectrodes arranged in an 8 × 8 layout grid embedded in a transparent glass substrate (59 recording electrodes, one internal reference electrode). The diameter of each electrode is 30 μm with an inter-electrode distance of 200 μm holding an impedance of <100 kΩ. The extracellular voltage transients induced by current flow through membranes of neurons were measured as local field potentials from spontaneous neuronal activity via the 60 microelectrodes. During the measurement, the MEA dish was taken out of the incubator and placed on the head stage of the recording system preheated to 37 °C and equilibrated for 2–5 min. To eliminate evaporation and contamination during the experiment, the MEA culture dishes were sealed with a removable semi-permeable membrane cover (ALA MEA–MEM–SHEET) secured with a glass ring (ALA Scientific Instruments Inc, Farmingdale, NY, USA) based on the method used in earlier work by Potter et al. [100]. The temperature of the head stage was controlled with an external heater unit (TC02, Multi-Channel Systems GmbH, Reutlingen, Germany) set to a constant temperature of 37 °C. Field potential streams were acquired through the amplification system from electrodes with a sampling rate of 100 kHz using MC Rack v3.7 software (Multi-Channel Systems GmbH, Reutlingen, Germany) digitized with a 60-channel A/D converter at a rate of 20 kHz. The number of spikes per minute and the spike rate was determined at 5 min intervals. The recordings were carried out without changing the medium in the MEA dishes. After the completion of the recordings, the medium was changed and the MEA dish with differentiated cells was returned to the incubator to be measured again at later time points to maintain consistency and reliability of the data and allow for self-comparison. The data from the recording exhibiting noise with asynchronous spike activity were identified and removed from the analysis.

### 4.11. Statistical Analysis

Six samples were set up for each condition in every experiment, and each experiment was repeated thrice. The data represent the mean ± SD for *n* = 6, analyzed using Microsoft Excel software (10, Microsoft, Redmond, WA, USA). The statistical significance of the differences between the different conditions and times were tested by two-way analysis of variance (ANOVA), followed by post–hoc analysis (Tukey’s test) using SPSS 24 software (24, IBM, Armonk, NY, USA), and *p ≤* 0.05 was considered statistically significant.

## 5. Conclusions

Our findings showed that compared to the three surfaces that we studied as substrata for the differentiation of NSCs, the CNC–Lys surface maximally supported the attachment and survival of rNSCs, allowing their differentiation into predominantly neurons. Importantly, CNC–Lys not only enhanced the maturity of neurons across the three weeks of culture, but also helped in driving the stem cells toward the formation of glutamatergic neurons that did not express much GABA or motor neuron markers. The differentiated cells formed connections and elicited electrical signals, confirmed by electrophysiological activity. In follow-up work, in vivo implantation of the differentiated neurons (on CNC–Lys) to explore their survivability is warranted, along with the profile of the genes expressed by the neurons differentiated on the CNC–Lys surface, to elucidate the cellular mechanism(s) involved in the enhanced neuronal differentiation observed on the CNC–Lys surface in the present study. In the future, we will study the modification efficiency of CNC with lysine by using varying amounts of EPH and the nanoparticle size of the modified materials. Then, we will use those surfaces to observe how the extent of CNC modification will affect NSC differentiation.

## Figures and Tables

**Table 1:** Cell counts (based on counting DAPI–stained cells) on each condition as the experiment progressed from one to three weeks (values expressed as mean ± SD).

Culture Substratum	Total Cells Plated/Dish	Cell Counts		
		Week 1	Week 2	Week 3
CNC	10,000	3450 ± 160.33	3374 ± 137.72	2561 ± 99.79
PDL	10,000	8531 ± 149.81	8188 ± 118.15	8150 ± 120.56
CNC–Lys	10,000	9423 ± 109.4	8329 ± 251.1	8199 ± 177.06

## Data Availability

The data are kept in the corresponding laboratories and would be available upon request.

## Supplementary Materials

The following are available online at https://www.mdpi.com/article/10.3390/jfb12040064/s1, Figure S1: XRD spectra of samples CNC (red spectrum) and CNC–Lys (purple spectrum), Figure S2: NSCs propagated well in cell culture showing the healthy, actively proliferating NSCs in our culture condition at 3 weeks, Figure S3: Representative images of mature neurons differentiated from rNSCs on CNC, and PDL and CNC–Lys surfaces. Neurons were labeled with MAPII neuronal marker for 1 (A, B, C), 2 (D, E, F) and 3 (G, H, I) weeks in culture. G1 and H1, I1 are images at higher magnification of cells cultured on CNC, PDL and CNC–Lys respectively. Neurons expressing βIII tubulin are green; cells expressing MAPII are red; and all the nuclei are counterstained blue by DAPI in all panels. The total population of cells were quantified for each experimental condition with CNC (A, D, G); PDL (B, E, H) and CNC–Lys (C, F, I) surfaces. The percentage of mature neurons was quantified (M) across 3 weeks on CNC, PDL and CNC–Lys surfaces. Scale bars represent 200 μm, Figure S4: Representative images of Local filed potentials of differentiated neurons from rNSCs on CNC, PDL and PDL and CNC–Lys surfaces show high frequency oscillations between (10–100 Hz) in vitro. Images acquired from 59 electrodes (including the reference electrode) from an MEA dish with recorded data. Representative phase contrast images (A, B, E, I: ×100 magnification) show NSCs differentiating post plating. The cells attached well and were nearby or in direct contact with the electrodes in the MEA chamber. (A,B,I) Filtered signals show extracellular local field potentials (spontaneous activity) on CNC (B), PDL (F) and CNC–Lys (J) coated surfaces with low pass filter cutoff frequency set at 100 Hz. Each square comprises the entire 300 s registration with a 50 ± 5 μV interval. (C, G, K) Representative trace on an expanded time scale showing characteristic extracellular local field potentials from the neurons differentiated on CNC (C), PDL (G) and CNC–Lys (K) coated surface. (D, H, L) Analysis of the data using Multi Channel Analyzer of the entire trace (300 s) reveals several peaks with varied frequencies and amplitudes.
