# Supplementary material for: Functionalized Nanocellulose Drives Neural Stem Cells toward Neuronal Differentiation
Source: J Funct Biomater. 2021 Nov 22;12(4):64. doi: 10.3390/jfb12040064 (PMC8628960; doi:10.3390/jfb12040064)
Supplement: Supplementary file 1 [file jfb-12-00064-s001.zip › jfb-1429387-supplementary.pdf]

Journal of *Functional Biomaterials*

MDPI

Supplementary

Functionalized Nanocellulose Drives Neural Stem Cells Toward Neuronal Differentiation

Sahitya Chetan Pandanaboina [1,†], Ambar B. RanguMagar [2,†], Krishna D. Sharma [1], Bijay P. Chhetri [2], Charlette M. Parnell [2], Jennifer Yanhua Xie [3,*], Malathi Srivatsan [1,*] and Anindya Ghosh [2,*]

1 Department of Biological Sciences and Arkansas Biosciences Institute, Arkansas State University, Jonesboro 72401, AR, USA; drpandana@gmail.com (S.C.P); krishna.sharma@smail.astate.edu (K.D.S.)
2 Department of Chemistry, University of Arkansas at Little Rock, Little Rock 72204, AR, USA; abrangumaga@ualr.edu (A.B.R.); bpchhetri@ualr.edu (B.P.C.); cmparnell15@yahoo.com (C.M.P.)
3 Department of Basic Sciences, New York Institute of Technology College of Osteopathic Medicine, Arkansas State University, Jonesboro 72401, AR, USA
* Correspondence: jennifer.xie@nyit.edu (J.Y.X.); msrivatsan@astate.edu (M.S.); axghosh@ualr.edu (A.G.); Tel.: +1-870-680-8877 (J.Y.X.); +1-870-897-6526 (M.S.); +1-501-916-6542 (A.G.)
† These authors contributed equally to this work.

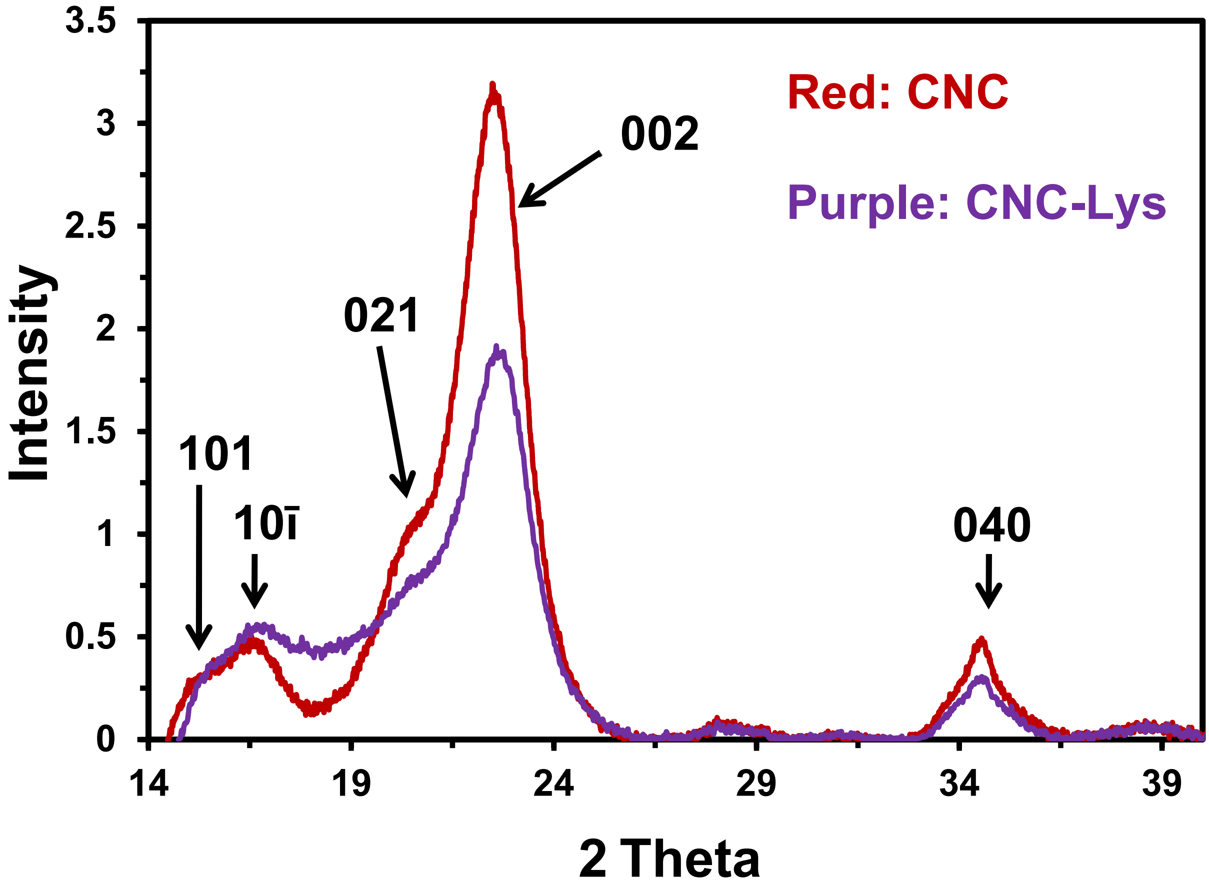

Figure S1. XRD spectra of samples CNC (red spectrum) and CNC–Lys (purple spectrum).

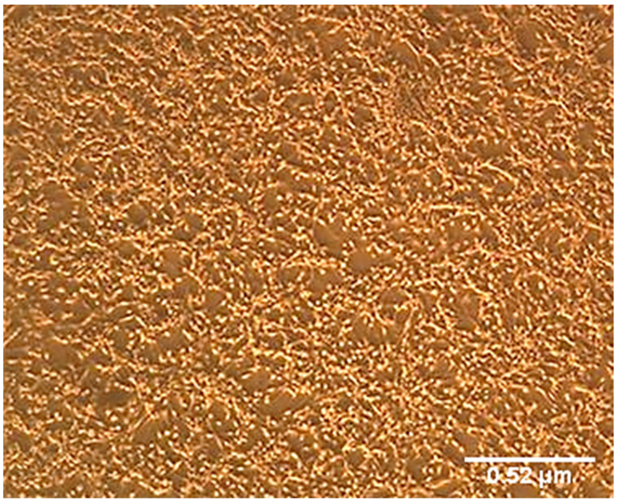

Figure S2. NSCs propagated well in cell culture showing the healthy, actively proliferating NSCs in our culture condition at 3 weeks.

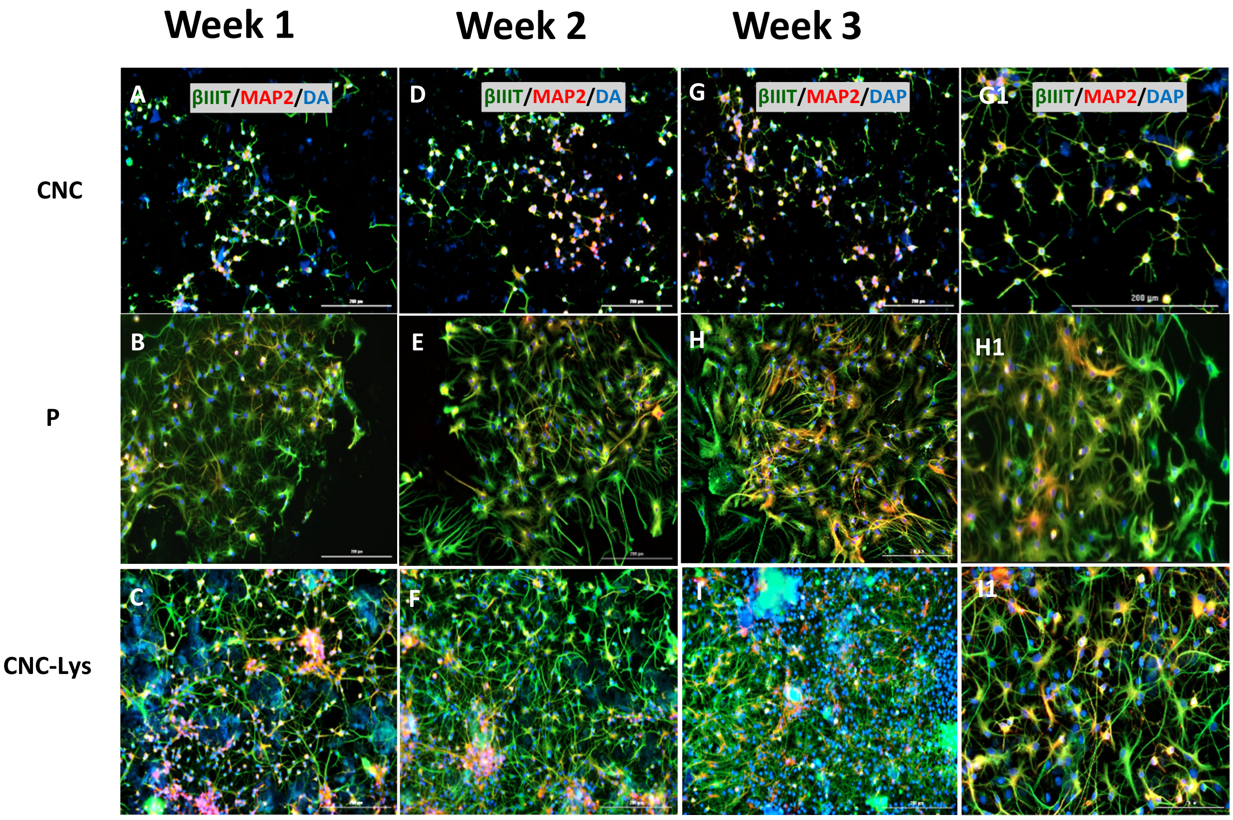

Figure S3. Representative images of mature neurons differentiated from rNSCs on CNC, and PDL and CNC–Lys surfaces. Neurons were labeled with MAPII neuronal marker for 1 (A, B, C), 2 (D, E, F) and 3 (G, H, I) weeks in culture. Figures G1 H1, I1 are images at higher magnification of of cells cultured on CNC, PDL and CNC–Lys respectively. Neurons expressing βIII tubulin are green; cells expressing MAPII are red; and all the nuclei are counterstained blue by DAPI in all panels. The total population of cells were quantified for each experimental condition with CNC (A, D, G); PDL (B, E, H) and CNC–Lys (C, F, I) surfaces. The percentage of mature neurons was quantified (M) across 3 weeks on CNC, PDL and CNC–Lys surfaces. Scale bars represent 200 µm.

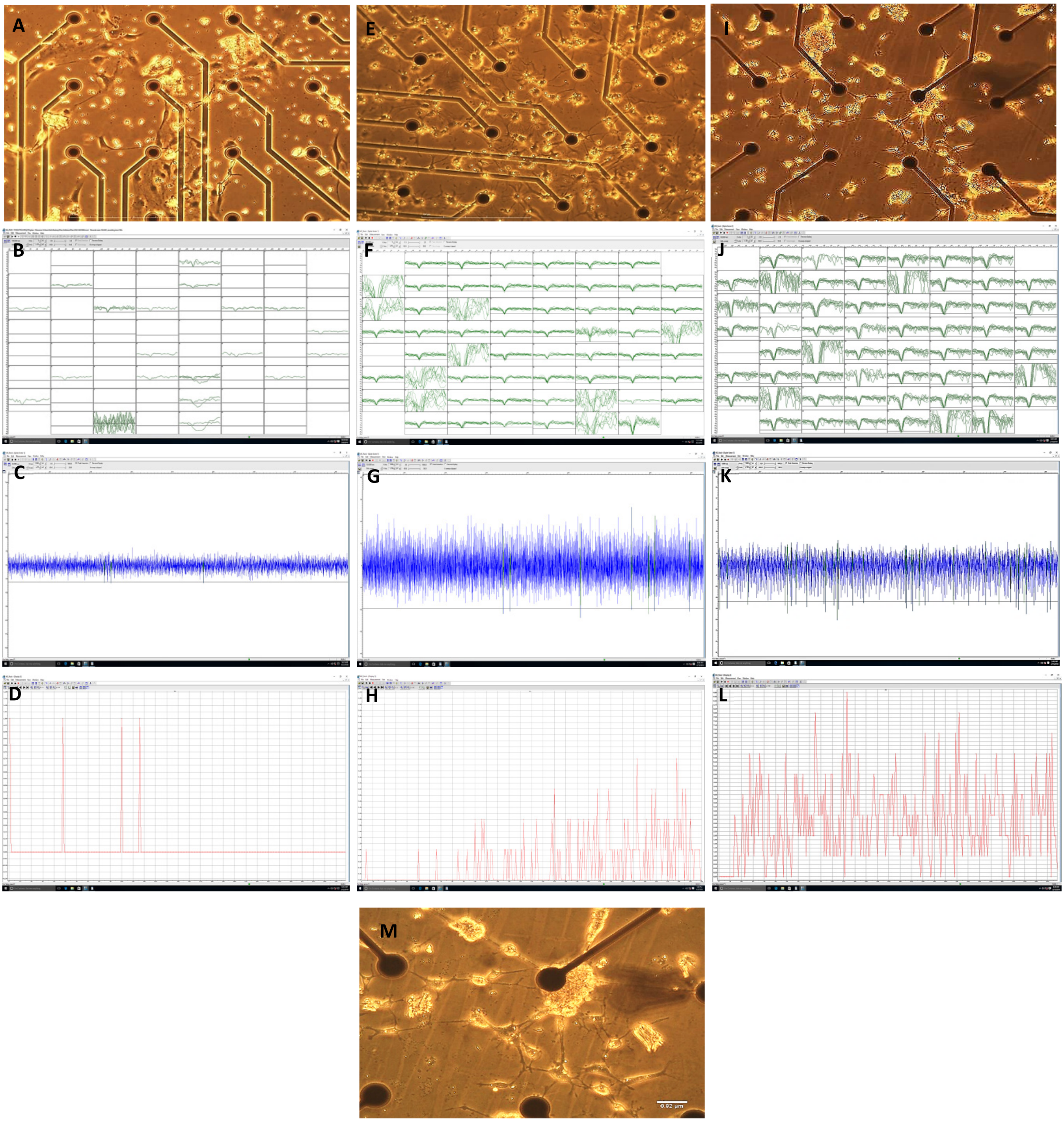

Figure S4. Representative images of Local filed potentials of differentiated neurons from rNSCs on CNC, PDL and PDL and CNC–Lys surfaces show high frequency oscillations between (10–100 Hz) *in vitro*. Images acquired from 59 electrodes (including the reference electrode) from an MEA dish with recorded data. Representative phase contrast images (A, B, E, I: ×100 magnification) show NSCs differentiating post plating. The cells attached well and were nearby or in direct contact with the electrodes in the MEA chamber. A,B,I) Filtered signals show extracellular local field potentials (spontaneous activity) on CNC (B), PDL (F) and CNC–Lys (J) coated surfaces with low pass filter cutoff frequency set at 100 Hz. Each square comprises the entire 300 sec registration with a 50 ± 5 µV interval. C, G, K) Representative trace on an expanded time scale showing characteristic extracellular local field potentials from the neurons differentiated on CNC (C), PDL (G) and CNC–Lys (K) coated surface. D, H, L) Analysis of the data using Multi Channel Analyzer of the entire trace (300s) reveals several peaks with varied frequencies and amplitudes. .
